# Using approximate Bayesian computation for estimating parameters in the cue-based retrieval model of sentence processing

**DOI:** 10.1016/j.mex.2020.100850

**Published:** 2020-03-03

**Authors:** Shravan Vasishth

**Affiliations:** University of Potsdam*,* Germany

**Keywords:** Bayesian parameter estimation, Prior and posterior predictive distributions, Psycholinguistics

## Abstract

A commonly used approach to parameter estimation in computational models is the so-called grid search procedure: the entire parameter space is searched in small steps to determine the parameter value that provides the best fit to the observed data. This approach has several disadvantages: first, it can be computationally very expensive; second, one optimal point value of the parameter is reported as the best fit value; we cannot quantify our uncertainty about the parameter estimate. In the main journal article that this methods article accompanies (Jäger et al., 2020, Interference patterns in subject-verb agreement and reflexives revisited: A large-sample study, Journal of Memory and Language), we carried out parameter estimation using Approximate Bayesian Computation (ABC), which is a Bayesian approach that allows us to quantify our uncertainty about the parameter's values given data. This customization has the further advantage that it allows us to generate both prior and posterior predictive distributions of reading times from the cue-based retrieval model of Lewis and Vasishth, 2005.

• Instead of the conventional method of using grid search, we use Approximate Bayesian Computation (ABC) for parameter estimation in the [4] model.
• The ABC method of parameter estimation has the advantage that the uncertainty of the parameter can be quantified.

## Graphical abstract

**Figure:**
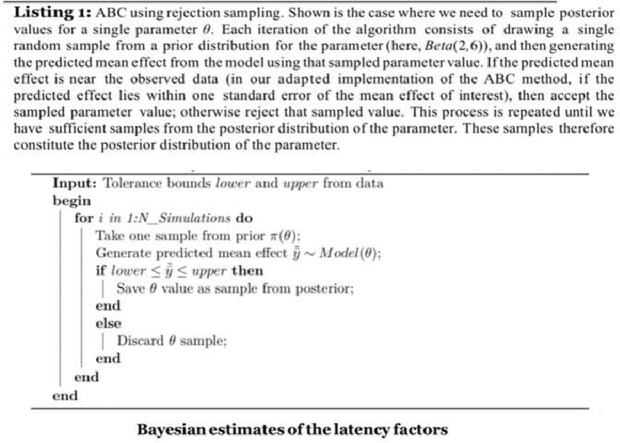

**Table:** Specification Table

Subject Area	*Psychology*
More specific subject area:	*Psycholinguistics*
Method name:	*Approximate Bayesian Computation using rejection sampling*
Name and reference of original method	Sisson, S. A., Fan, Y., & Beaumont, M. [5]. *Handbook of approximate Bayesian computation*. Chapman and Hall/CRC.
Resource availability	https://osf.io/reavs/

## Method details

This paper is a companion to Jäger, Mertzen, Van Dyke, and Vasishth [1], and shows how we estimate the latency factor parameter in the cue-based retrieval model of Engelmann, Jäger, and Vasishth [2], when evaluating the model's predictions to the observed data from Dillon, Mishler, Sloggett, and Phillips [3] and our larger-sample replication attempt [1]. The source code and data associated with the methods reported here and the paper by Jäger et al. [1] are available from https://osf.io/reavs/. More general background regarding the model is provided in [8].

## The cue-based retrieval model of [2]

The Engelmann et al. model of sentence processing is a simplified version of the Lisp-based model described in Lewis and Vasishth [4]. This simplified version is written in R and abstracts away from the individual incremental parsing steps of the original model, and focuses instead only on the retrieval time and retrieval accuracy computations, given some retrieval cues and candidate chunks in memory that could match the retrieval cues.

Table 1 shows the parameter values used in the recent large-sample model evaluation (approximately 100 published reading experiments) of the cue-based retrieval model described in Engelmann et al. [2]. Here, we follow the practice that was adopted in Lewis and Vasishth [4], of holding all the parameters constant to their default value. The only exception is the latency factor parameter, which scales retrieval time to the millisecond reading time scale. The reason for holding the parameters constant is to avoid overfitting to the particular data being considered.

**Table 1:** *Model parameters, their default values, and the values used in the simulation of the studies discussed in*[2]*.*

Parameter	Name	Default	Simulation
*F*	latency factor	0*.*2	[0*.*1*,* 0*.*25]
*f*	latency exponent	1	1
*τ*	retrieval threshold	−1*.*5	−1*.*5
*d*	decay rate	0*.*5	0*.*5
*ANS*	activation noise	0*.*2	0*.*2
*MAS*	maximum associative strength	1	1*.*5
*MP*	mismatch penalty	1	0*.*25
*β*	base-level activation	0	0

## Bayesian parameter estimation

Here, we provide some of the background needed to understand the parameter estimation approach described below. In the Bayesian modeling framework, given a vector of data *y* and a vector of model parameters *θ* that have prior distributions *p*(*θ*) defined on them, a likelihood function for the data *p*(*y* | *θ*) and the priors allow us to compute the posterior distribution of the parameters given the data, *p*(*θ* | *y*). This is possible because of Bayes’ rule, which states that the posterior is proportional to the likelihood times the prior:

p(θ|y)∝p(y|θ)p(θ)

The posterior distributions of parameters are generally computed using Monte Carlo Markov Chain methods. Examples are Gibbs sampling, Metropolis-Hastings, and (more recently) Hamiltonian Monte Carlo. The likelihood and the priors together constitute the model, which we will call M hereafter. Given a particular model M, one important question is: how can we derive the predictions of the model? The model makes two kinds of predictions: a priori predictions, before any data have been taken into account; and a posteriori predictions, after the data have been taken into account. The distributions of these two kinds of predictions are called *prior predictive distributions*, and *posterior predictive distributions*, respectively.

The prior predictive distribution can be computed by drawing random samples of the parameters $\tilde{\theta }$ from *p*(*θ*), and then using these values to simulate data $\tilde{y}$ from the likelihood $p(y|\tilde{\theta })$.

The posterior predictive distribution *p*(*y_pred_* | *y*) can be computed once we have the posterior distribution of the parameters, *p*(*θ* | *y*).

$$p\left(y_{pred}|y\right)=\int p\left(y_{pred}|\theta \right)p\left(\theta |y\right)d\theta$$

An important point to note here is that we are conditioning *y_pred_* only on *y*. We do not condition on the unknown parameters *θ*; we simply integrate these unknown parameters out. This allows us to take the uncertainty of the posterior distributions of the parameters into account, giving us more realistic estimates of the predictions from the model. Contrast this with a situation where we condition on, e.g., maximum likelihood estimates of the parameters; that is, we condition on a point value, not taking the uncertainty of that estimate into account.

## Approximate Bayesian computation

Approximate Bayesian Computation (ABC) is a method for estimating posterior distributions of parameters in a model. ABC is useful when Bayes’ rule cannot be employed to draw samples from the posterior distributions; this situation arises when the generative model cannot be easily expressed as a likelihood function. For extensive treatments of the theory and practical aspects of ABC, see Sisson, Fan, and Beaumont [5], Palestro, Sederberg, Osth, Van Zandt, and Turner [6]. The algorithm that we adapt for our purposes here is rejection sampling; see Listing 1 below for pseudo-code describing the algorithm.

**Listing 1:**
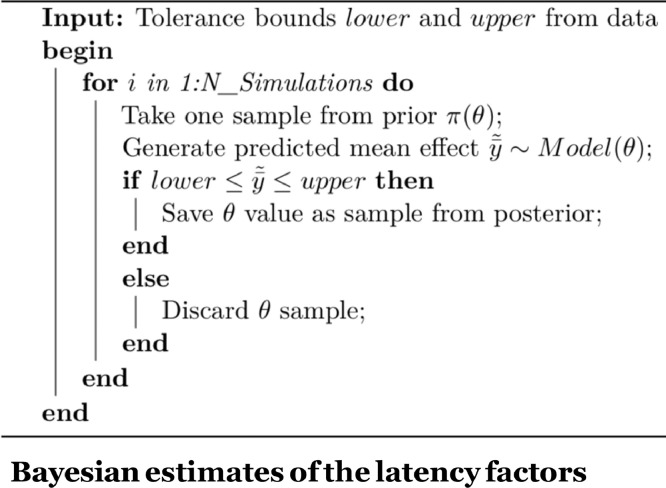
ABC using rejection sampling. Shown is the case where we need to sample posterior values for a single parameter *θ*. Each iteration of the algorithm consists of drawing a single random sample from a prior distribution for the parameter (here, *Beta*(2, 6)), and then generating the predicted mean effect from the model using that sampled parameter value. If the predicted mean effect is near the observed data (in our adapted implementation of the ABC method, if the predicted effect lies within one standard error of the mean effect of interest), then accept the sampled parameter value; otherwise reject that sampled value. This process is repeated until we have sufficient samples from the posterior distribution of the parameter. These samples therefore constitute the posterior distribution of the parameter.

**Step 1: Define a prior for the parameter**

**Fig. 1:**
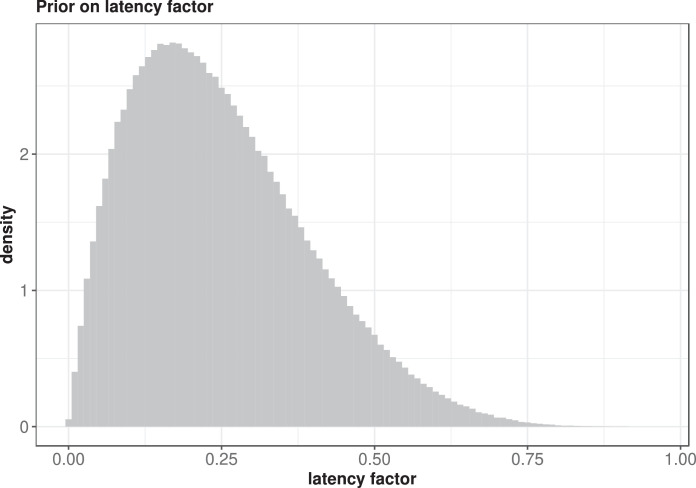
A Beta(2,6) prior on the latency factor.

We begin by defining a prior distribution on the latency factor in the cue-based retrieval model. Several priors can be considered: a Uniform prior or a Beta prior are examples. For illustration, we use the Beta(2,6) prior. As shown in Fig. 1 this is a relatively uninformative prior which downweights very small and very large values of the latency factor parameter.

**The estimates from data for ungrammatical conditions.** In the ungrammatical conditions of the Dillon et al. [3] data, the estimate of the interference effect in agreement conditions is −60 ms, Credible interval (CrI) [−112, −5] ms. Taking a normal approximation, this implies an effect coming from the distribution *Normal*(−60*,* 33^2^). Similarly, the estimate of the interference effect in reflexive conditions is −18 ms, CrI [−72, 36] ms, which corresponds approximately to the *Normal*(−18*,* 27^2^).

We can use these normal approximations to define a lower and upper bound for the ABC algorithm: one standard deviation about the observed mean. The acceptance criterion of the ABC algorithm is that the predicted value generated by the model lies within one standard deviation of the sample mean from the data. One standard deviation is chosen here just to reasonably constrain the range of acceptable values. If we had chosen two standard deviations as a criterion, this would lead to a more broadly distributed posterior for the parameter of interest (the latency factor), and if we had chosen half a standard deviation, we would obtain a more narrowly distributed posterior. The predicted effects from the model would accordingly be more broadly (two SDs) or more tightly (half an SD) distributed. The qualitative predictions from the model do not change.

In the Jäger et al. [1] data, the estimate of the interference effect in agreement conditions is −22 [−46, 3], which can be approximated by the *Normal*(−22*,* 13^2^). The estimate in reflexive conditions is −23 [−48, 2], which can be approximated as the *Normal*(−23*,* 13^2^).

**Step 2: Compute posterior distributions of the latency factor using ABC rejection sampling**

**Fig. 2:**
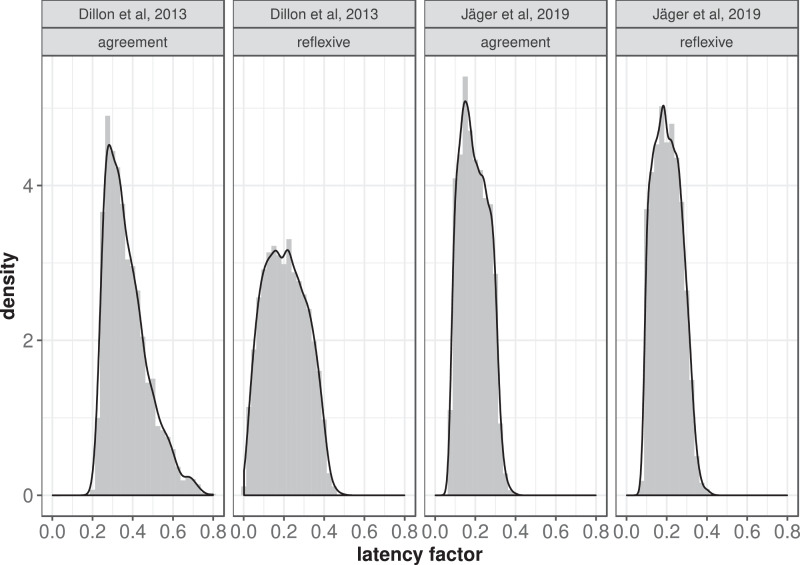
The posterior distributions of the latency factor parameters for agreement and reflexive conditions using the original [3] data (40 participants, 48 items) and our own [1] replication data (181 participants, 48 items).

Fig. 2 shows the posterior distributions of the latency factor parameter for ungrammatical agreement and reflexive conditions in Dillon et al. [3] and Jäger et al. [1]. The estimates for the Dillon et al. [3] data-set have wider uncertainty than those for Jäger et al. [1] because the uncertainty of the interference effects in the data is relatively large.

**Step 3: Generate posterior predicted data**

**Fig. 3:**
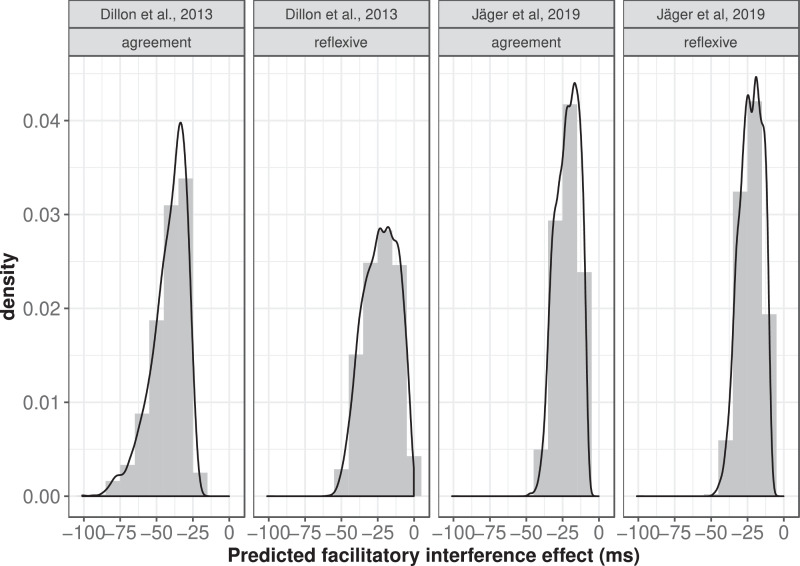
The posterior predictive distributions of the facilitatory interference in ungrammatical agreement and reflexive conditions, derived using the posterior distributions of the latency factor parameter.

Having estimated the posterior distributions of the latency factor for the two data-sets in the two conditions (agreement and reflexives), we can now generate posterior predicted data from the model. We use the posterior distributions of the latency factor to generate the posterior predictive distribution of the interference effect in these experimental conditions. These posterior predictive distributions are shown in Fig. 3.

The ABC method can be generalized using other, more efficient sampling approaches (e.g., Metropolis-Hastings) to sample the posterior from more than one parameter. The method is computationally expensive but the advantages afforded by taking parameter uncertainty into account in the predictions is very valuable.

## Concluding remarks

In closing, the ABC method is a powerful tool for parameter estimation in models like the cue-based retrieval model, which cannot be easily expressed as a likelihood. To our knowledge, [7], were the first to adopt ABC in estimating parameters in an ACT-R model. Although this approach has not yet been widely adopted in the ACT-R modeling community, ABC holds great promise for modeling researchers because it allows us to take parameter uncertainty into account when evaluating model predictions. This will yield more realistic predictions than using point values for parameters.
